# The Balance between Life and Death of Cells: Roles of Metallothioneins

**Published:** 2007-02-07

**Authors:** Allan Evald Nielsen, Adam Bohr, Milena Penkowa

**Affiliations:** Section of Neuroprotection, Centre of Inflammation and Metabolism.The Panum Institute, Faculty of Health Sciences, University of Copenhagen, Denmark

**Keywords:** Metallothionein, cell survival, apoptosis, metal regulation, free radicals

## Abstract

Metallothionein (MT) is a highly conserved, low-molecular-weight, cysteine-rich protein that occurs in 4 isoforms (MT-I to MT-IV), of which MT-I+II are the major and best characterized proteins.

This review will focus on mammalian MT-I+II and their functional impact upon cellular survival and death, as seen in two rather contrasting pathological conditions: Neurodegeneration and neoplasms. MT-I+II have analogous functions including: 1) Antioxidant scavenging of reactive oxygen species (ROS); 2) Cytoprotection against degeneration and apoptosis; 3) Stimulation of cell growth and repair including angiogenesis/revascularization, activation of stem/progenitor cells, and neuroregeneration. Thereby, MT-I+II mediate neuroprotection, CNS restoration and clinical recovery during neurodegenerative disorders. Due to the promotion of cell survival, increased MT-I+II levels have been associated with poor tumor prognosis, although the data are less clear and direct causative roles of MT-I+II in oncogenesis remain to be identified.

The MT-I+II molecular mechanisms of actions are not fully elucidated. However, their role in metal ion homeostasis might be fundamental in controlling Zn-dependent transcription factors, protein synthesis, cellular energy levels/metabolism and cell redox state.

Here, the neuroprotective and regenerative functions of MT-I+II are reviewed, and the presumed link to oncogenesis is critically perused.

## Introduction

Metallothionein (MT) is a low-molecular-weight (6–7 kDa), nonenzymatic and metal-binding protein that is well conserved in the animal kingdom. Mammalian MT constitutes a superfamily of proteins that all belong to class I MTs, of which four isoforms (MT-I, MT-II, MT-III and MT-IV) have been identified so far. They are all encoded by a family of genes located in humans on chromosome 16q13, where at least 16 MTs have been identified, and these were primarily grouped into isoforms MT-I and -II (MT-I+II) (Karin et al. 1984; Vallee, 1995; Kägi and Kojima, 1987; Vasak, 2005).

Although it is not consistently regarded as a fifth MT isoform, the testis-specific protein tesmin was described as a MT-like protein and named MT-like 5. But time will show if and how many additional MT isoforms exist in mammals (Vallee, 1995; Dincer et al. 1999; Klaassen et al. 1999; Ghoshal and Jacob, 2001; Mocchegiani et al. 2005; Vasak, 2005).

MT-I-II are the best characterized MTs and have been known for almost 50 years since Margoshes and Vallee (1957) identified a cadmium-containing protein in equine kidneys. In the 1990’s, MT-III and MT-IV were found, but these isoforms are poorly characterized and the data have been rather divergent for MT-III, while MT-IV has only been studied in the skin epidermis (Hidalgo et al. 2001; Vasak, 2005; Aschner and West, 2005). However, the data indicate that MT-III roles in the brain are significantly different from those of MT-I+II (Chung and West, 2004; Vasak, 2005; Penkowa et al. 2006b).

This review will only describe the MT-I+II proteins, which are regulated and expressed coordinately (Searle et al. 1984), and in mammals they have analogous biological functions (Chung and West, 2004; Penkowa et al. 2006b).

## MT-I+II Structural Properties

The mammalian MT-I+II are composed of 61 and 62 amino acids, respectively, which are characterized by a lack of aromatic amino acids and histidine as well as high contents of cysteines (20 out of 61 amino acids are cysteines ~ 30%) that form metal thiolates by their sulfhydryl groups, and also, MT-I+II contain up to 14% lysine plus arginine (Vallee, 1995; Klaassen et al. 1999; Ghoshal and Jacob, 2001; Hidalgo et al. 2001; Mocchegiani et al. 2005; Vasak, 2005).

**Figure 1:**
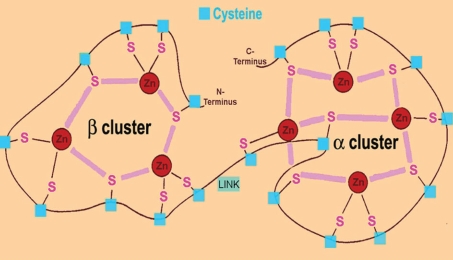
Drawing of the mammalian MT-II protein showing the two metal-thiolate clusters (C-terminal α-domain and N-terminal β-domain) including the 20 cysteine residues and their sulphur atoms (S), which bind to divalent or monovalent cations (in this case Zn). The domains are linked by a short peptide containing amino acid residues 30–32 in mammalian MT-II LINK. In the β-domain, 3 divalent or 6 monovalent metal ions are coordinated, while in the α-domain 4 divalent or 6 monovalent cations can be bound.

The cysteine residues appear in conserved Cys-X_n_-Cys motifs, where X is any other amino acid than cysteine (Vallee, 1995; Klaassen et al. 1999; Ghoshal and Jacob, 2001; Vasak, 2005). The cysteine thiol (SH-)group of the metal-free protein (apothionein) can complex 7 divalent or 12 monovalent metal ions per MT molecule (Fig. 1), and this binding of metals is required for the folding and the final three-dimensional conformation of MT resulting in a molecule with two native metal thiolate clusters residing in two separate globular domains, the C-terminal α-domain and N-terminal β-domain linked by a short bridging region (Vallee, 1995; Klaassen et al. 1999; Ghoshal and Jacob, 2001; Maret, 2002; Romero-Isart and Vasak, 2002; Blindauer and Sadler, 2005; Vasak, 2005). The metal thiolate clusters of MT-I+II also confers molecular stability and reduces significantly the susceptibility to degradation (Klaassen et al. 1994; Miles, 2000). This is also reflected by the fact that apothionein is rather unstable and is rapidly degraded and excreted, while in rodents Zn-loaded MT has a halftime of up to 18–22 hours (Jiang et al. 1998; Klaassen et al. 1994; Miles et al. 2000).

The three-dimensional structure was revealed decades ago and in principle by using X-ray crystallography and NMR spectroscopy. The described isoform was mammalian MT-II loaded with divalent metal ions, which by both the NMR and crystal structure showed similar metal thiolate cluster conformation and practically an identical polypeptide folding (Romero-Isart and Vasak, 2002; Vasak, 2005). Additional NMR studies validated that binding of either Zn or/and Cd ions would both lead to an identical MT conformation. Besides, the NMR solution structure of mouse MT-I versus MT-II showed that these isoforms display a high degree of similarity, as the only significant difference between MT-I and MT-II was the level of β-domain flexibility (Romero-Isart and Vasak, 2002; Vasak, 2005).

The high amounts of cysteine including sulphur allow MT to bind diverse metals such as (but not restricted to) Zn, Cu, Cd, Hg, Pb, Ni and Co (Haq et al. 2003; Kelly and Palmiter, 1996; Klaassen et al. 1999; Vasak, 2005). The 20 cysteine residues are distributed in the MT domains, showing 11 cysteines in the α-domain (amino acid residues 33–61) that can bind four divalent or six monovalent metals; and 9 cysteines in the β-domain (amino acids 1–29) capable of binding three divalent or six monovalent metals (Klaassen et al. 1999; Hidalgo et al. 2001; Romero-Isart and Vasak, 2002; Vasak, 2005).

## MT-I+II Functional Aspects

During physiological conditions, an essential role of MTs still remains elusive despite extensive studies, however it is generally acknowledged that they are important for some intracellular processes including the ability to regulate metals including donation and transport of essential metals to other proteins and enzymes (as reviewed by Davis and Cousins, 2000; Miles, 2000; Palmiter, 1998).

Interest in the role of MT-I+II is growing due to their possible roles in cell survival versus death, and particularly in the brain, MT-I+II may provide new therapeutic agents to be used against neurode-generative diseases (for review see: Aschner and West, 2005; Chung and West, 2004).

These roles were originally identified in the brain by using genetically MT-modified mice in combination with an experimental model of brain injury (Penkowa et al. 1999). The application of pathology is today considered as a prerequisite in order to detect any major changes in vivo of MT-I+II genetic deficiency (for review see: Hidalgo et al. 2002; West et al. 2004). Thus, MT-I+II knockout (MT-KO) mice that are not subjected to a brain injury appear rather unaffected and develop normally (Michalska and Choo, 1993). Accordingly, in healthy physiological conditions, MT-I+II are considered to be rather negligible or have rich compensatory backup molecules. However, the MT-KO mice were more susceptible to cadmium toxicity relative to wildtype control mice (Masters et al. 1994).

In case a brain injury is applied, it is evident that MT-I+II have neurotrophic and protective actions enhancing neuronal survival, brain tissue remodeling and repair (Aschner and West, 2005; Penkowa, 2006). Even if the precise molecular mechanisms of MT-I+II actions are not yet clarified, some factors and intracellular processes are known to be altered or regulated by MT-I+II, and these include (but are not restricted to): Metal ion homeostasis, scavenging of ROS, and inhibition of pro-apoptotic mechanisms like caspases and p53. These aspects of MT-I+II biology are the focus of this review.

However, MT-I+II exert additional and rather wide-ranging functions in pathology and consequently engage in processes such as protein-protein and protein-nucleotide interactions, regulation of transcription factors, mitochondrial respiration, thermogenesis, body energy metabolism, angiogenesis, cell cycle progression, and cell differentiation. Some of the MT-I+II molecular mechanisms and signaling pathways have been identified and as they have recently been reviewed by different groups, we kindly refer to these (Aschner and West, 2005; Chung and West, 2004; Penkowa, 2006; Vasak, 2005; West et al. 2004).

### Metal Ion Homeostasis

In the brain, dysregulation of essential metals is associated with a number of diseases and neuropathologies including stroke, epilepsy, traumatic brain injury, neurodegenerative disorders like Parkinson’s disease and Alzheimer’s disese (Andrews, 2000; Frederickson et al. 2004; Hidalgo et al. 2001, 2002). MT-I+II function as a metal ion buffer as they can release the bound metal ions, whereby MTs have important roles in metal homeostasis. This is most likely to contribute to the described MT-I+II cytoprotection (Kägi and Kojima, 1987; Kelly and Palmiter, 1996; Mocchegiani et al. 2005; Penkowa, 2006).

By acting both as a donor and acceptor of essential metals Zn and Cu, MT-I+II can direct Zn- and Cu-dependent proteins, enzymes, and transcriptions factors in cells (Blindauer and Sadler, 2005; Davis and Cousins, 2000; Ghoshal and Jacob, 2001; Kelly et al. 1996; Maret, 2002; Romero-Isart and Vasak, 2002; Ye et al. 2001).

In mammals, the Zn regulation by MT-I+II is likely of major importance, since Zn is essential for many cellular functions (Maret, 2002; Mocchegiani et al. 2005; Vasak, 2005; Ye et al. 2001). However, a deficiency or overload with Zn is cytotoxic, and Zn-related diseases like acrodermatitis enteropathica and the lethal milk syndrome may occur. Especially the brain is susceptible to dysregulated Zn concentrations that cause severe neuronal damage (Kelly et al. 1996; Frederickson et al. 2004). When Zn is lowered such as seen during a dietary insufficiency, MT-I+II will usually release the Zn and thereby become degraded leading to significantly decreased MT-I+II expression levels (Hidalgo et al. 2002), not only in peripheral tissues but also in the brain tissue. During conditions with excess Zn in the brain, MT-I+II mRNA and protein are increased in order to bind and neutralize the metals (Kelly et al. 1996; Miles et al. 2000; Hidalgo et al. 2002; Haq et al. 2003).

### Metal Transfer and Redox Potential

Moreover, MT-I+II can transfer Zn to other metal-containing molecules like enzymes, antioxidant factors, Zn-finger proteins and transcription factors, which also are essential for several signaling pathways and for the cell fate (Frederickson et al. 2004; Klaassen et al. 1999; Maret, 2002; Maret, et al. 2002; Ye et al. 2001). This may occur directly by protein-protein interactions leading to a direct transfer of Zn from MT-I+II to mitochondrial aconitase (Feng et al. 2005).

Metals are also released from the thiolate clusters when MT-I+II are oxidized by constitutive, mild pro-oxidant factors such as glutathione disulfide (GSSG) or selenium compounds (Ye et al. 2001; Maret, 2002; Khatai et al. 2004). In the cells, disulfides such as GSSG can oxidize MT proteins leading to an immediate Zn release, whereas the glutathione (GSH) reduces the protein leading to a reuptake of the available Zn (Chen and Maret, 2001).

In fact, the glutathione (GSH)/GSSG redox pair can cooperate with MT/apothionein in order to coordinate the uptake and release of metals from the MT thiolates (Chen and Maret, 2001; Romero-Isart and Vasak, 2002). Another contributor to the release of metals from MTs is reactive oxygen and nitrogen species (ROS), which is part of the MT-I+II antioxidant mechanism of action. In general, ROS attack and oxidize the metal-thiolate clusters, and subsequently MT-I+II release their metals, which may be complexed again in case of reductive conditions and if the proper metals are provided (Romero-Isart and Vasak, 2002). However, it has also been reported by Feng et al. (2006) that MT-I+II can form intramolecular disulfide bonds in vivo, which increase after the release of the metal ions.

### Scavenging of Free Radicals

Oxidative stress refers to the cytotoxic consequences of a mismatch between the formation of ROS and the ability of the cells to produce antioxidants. During stressful and/or pathological conditions, pro-inflammatory responses and particularly activated leukocytes increase ROS generation significantly, and accordingly, inflammation is regularly followed by oxidation and/or nitration of lipids, proteins, DNA and carbohydrates (Allan and Rothwell, 2003; Espejo et al. 2005; Hidalgo et al. 2002; Mhatre et al. 2004; Penkowa and Hidalgo, 2001, 2003; Penkowa et al. 2005). Oxidative stress is a major inducer of apoptotic cell death and thus has major pathogenic roles in most degenerative disorders (Allan and Rothwell, 2003; Andrews, 2000; Hidalgo et al. 2002). Also, generation of oxidative stress is the mode of action of most anti-cancer therapies and cytotoxicity (Kondo et al. 1997; Klaassen et al. 1999; Theocharis et al. 2003).

In the 1980’s, Thornalley and Vasak, (1985) showed in a cell free system that MT-I is an efficient ROS scavenger that inhibits ROS-induced cyto- and nuclear toxicity more effectively than proteins 10–50-times its molecular weight. Since then, numerous in vitro studies have confirmed the MT-I+II antioxidant effects and also, it was shown that MT-I+II scavenge ROS with higher molar effectiveness than glutathione (Abel and de Ruiter, 1989; Cai and Cherian, 2003).

By using genetically modified cells, it became clear that MT-I overexpression protects against ROS cytotoxicity and vice versa in MT-I+II deficient cell cultures (Kondo et al. 1997; Klaassen et al. 1999; Wanpen et al. 2004).

**Figure 2:**
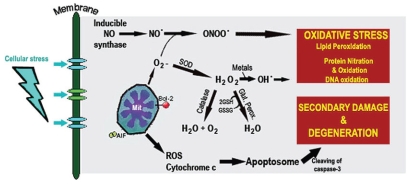
**Oxidative Stress and Secondary Damage.** ROS scavengers MT-I+II inhibit oxidative stress, in part due to their metal ion release. MT-I+II also counter apoptosis directly, e.g. through inhibition of cytochrom c leakage and caspase-3 activation.

As shown in animal models of various brain disorders, MT-I+II can also inhibit in vivo oxidative stress efficiently, where MT-I+II prevent ROS-mediated toxicity such as lipid peroxidation, protein tyrosine nitration due to peroxynitrite, and NO formation, which ultimately lead to neuronal degeneration and apoptotic cell death (Ebadi et al. 2005a,b;Gong and Elliott, 2000;Hidalgo et al. 2001, 2002; Nagano et al. 2001; Penkowa et al. 2006a,b; West et al. 2004). This alone, makes the MT-I+II antioxidant actions have a high impact upon brain dysfunctioning and cell death, but as described below, MT-I+II also have direct anti-apoptotic actions (Fig. 2), which become significantly important during CNS pathologies and in case of tumor growth including resistance to anti-cancer therapy.

From a number of in vitro and in vivo studies, it was shown that MT-I+II readily react with hydroxyl, superoxide, and nitric oxide; while the MT-I+II thiol groups can bind peroxynitrite anion and peroxynitrous acid (Aschner and West, 2005; Penkowa, 2006; Romero-Isart and Vasak, 2002). In yeast, increased MT-I+II levels were suggested to functionally compensate for deficiency of Cu/Zn-super oxide dismutase (Cu/Zn-SOD) in the defense against oxidative stress (Tamai et al. 1993). Moreover, MT-I+II induction may antagonize the deleterious effects of oxidative stress on catalase (Haidara et al. 1999).

In case of glutathione depletion, MT-I+II enrichment of cells can confer profound resistance towards oxidative damage (Min et al. 2005). As described above, MT-I+II may donate and accept metals from the GSSG/GSH system, and also during oxidative stress, MT-I+II interact with this system in order to inhibit GSH depletion (Jiang et al. 1998; Haidara et al. 1999). In this regard, it was reported that the α- and β-domains of MT-I+II may react differently to ROS exposure, even if the two domains appear rather similar. Hence, the N-terminal β-domain of MT-I would rapidly release all the complexed metals and form disulfide bonds after exposure to NO, while in contrast the C-terminal α-domain remained unaffected (Romero-Isart and Vasak, 2002; Feng et al. 2005). This indicates that the MT-I+II biological reactions are complex and deserve further attention, with the purpose of advancing the understanding of its functional properties.

### Inhibition of Apoptosis

Numerous in vitro, in vivo and human studies have shown anti-apoptotic actions of MT-I+II, although the actual mechanisms of action remain to be fully clarified.

First, MT-I+II are metal ion chelators, and they have significant antioxidant and anti-inflammatory actions, which may mediate the anti-apoptotic actions of MT-I+II (Aschner and West 2005; Penkowa et al. 2006). As pro-inflammatory responses including neurotoxic cytokines like interleukins and tumor necrosis factor-α (TNFα) can induce neuronal apoptosis per se, the MT-I+II reduction of brain inflammation may contribute to their protective effects.

However, it is not likely that these effects of MT-I+II provide all the answers, and particularly as mounting data have shown direct anti-apoptotic actions of MT-I+II (Chung et al. 2003; Køhler et al. 2003).

MT-I+II interact with a range of molecular pathways that directly or indirectly regulate the apoptotic cascade. A number of experiments have focused on the MT-I+II inhibition of the mitochondrial cytochrome-c release and the activation of caspase-3 (Fig. 2) (Ceballos et al. 2003; Cherian and Apostolova, 2000; Gong and Elliott, 2000; Giralt et al. 2002; Nagano et al. 2001; Penkowa et al. 2004,^2005^; Stankovic, 2005). The cytochrome-c outflow from the mitochondria is critical as an initiator of ROS-triggered formation of the apoptosome; which is followed by pro-caspase-3 cleavage generating active caspase-3, the executioner of the apoptotic death process (Potashkin and Meredith, 2006). During pro-apoptotic conditions, MT-I+II cause a significant reduction in the cytochrome-c leakage into the cytoplasm and reduced levels of caspases (e.g. caspase-1 and -3), although the precise molecular mechanism of action has not been clarified (Carrasco et al. 2000; Cherian and Apostolova, 2000; Espejo et al. 2005; Gong and Elliott, 2000; Giralt et al. 2002; Nagano et al. 2001; Penkowa et al. 2004, 2005; Penkowa and Hidalgo, 2001; Stankovic, 2005).

Another key inducer of apoptosis is the tumor suppressor protein p53, which is efficiently countered by MT-I+II by means of a direct interaction as shown by Ostrakhovitch et al. (2006). Hence, MT-I+II cause a p53-null state that accelerates tumor cell growth and survival (Ostrakhovitch et al. 2006).

Furthermore, nucleotides ATP and GTP are ligands that bind to MT-I+II (Jiang et al. 1998; Maret, 2002; Maret et al. 2002; Vallee, 1995) leading to changes in MT-I+II structural and functional properties (Maret et al. 2002). Also, MT-I+II and ATP levels are correlated, which in itself could indicate a role in cell loss or survival, since ATP depletion is part of the apoptotic signal cascade (Aschner and West, 2005; Ebadi et al. 2005a). The link between MT-I+II and ATP could also indicate other actions, such as a recently reported stabilization and restoration of the ageing mitochondrial genome by MT-I+II (Ebadi et al. 2005a), as well as the MT-I+II roles in energy balance and metabolism as mediated by interactions with mitochondrial factors like m-aconitase (Beattie et al. 1998; Haq et al. 2003; Ye et al. 2001).

Another possible anti-apoptotic mechanism of MT-I+II could be the reported interaction with transcription factor nuclear factor KappaB (NFκB), as MT-I+II control the cellular concentration and activity level of NFκB, which is implicated in both cell death and survival (Carrasco et al. 2000; Crowthers et al. 2000).

In a dose-dependent manner, MT-I+II also increase de novo synthesis and expression levels of common proto-oncogenes (e.g. c-myc), apoptosis-inhibitory genes (e.g. bcl-2) and a number of growth/trophic factors (like FGF, TGFβ, VEGF, BDNF, GDNF, NTs) that are all well-known as promoters of cell survival (Cherian and Apostolova, 2000; Ebadi et al. 2005b; Miles et al. 2000; Mocchegiani et al. 2005; Penkowa, 2006; Tekur and Ho, 2002)

According to these functions, degeneration and neuron loss are all more severe in MT-I+II deficient mice (MT-I+II knock-out (MT-I+IIKO) mice) than in wildtype controls, whilst the disorders are efficiently diminished by transgenic MT-I overexpression and exogenous MT-I or MT-II treatment (Aschner and West, 2005; Penkowa et al. 2006a,b).

## MT-I+II Perspectives

### Neurodegeneration

Brain pathology and neurological disorders are followed by acute pro-inflammatory responses of microglia/macrophages and lymphocytes secreting cytotoxic cytokines, tissue digesting enzymes, complement, and ROS (Penkowa et al. 2006a, b; Potts et al. 2006). In addition, increased metal ions with potential neurotoxic actions are seen in the damaged brain tissue, which can be due to a number of parameters such as cytolysis, pH changes, dysregulation of metal-binding factors, and breakdown of the blood-brain barrier (Andrews, 2000; Frederickson et al. 2004; Kelly et al. 1996).

Though inflammatory reactions are necessary for the brain tissue repair process, they also mediate collateral and delayed (secondary) damage to the brain. Particularly ROS are evidently implicated in secondary damage including neurodegeneration and ultimately apoptotic cell death (Allan and Rothwell, 2003; Mhatre et al. 2004; Potashkin and Meredith, 2006). ROS are particularly toxic in the brain, as the microenvironment is characterized by low antioxidant capacity and high content of lipids (myelin) that are readily oxidized and/or peroxidized (Penkowa et al. 2004, 2005; Potashkin and Meredith, 2006; Potts et al. 2006). However, if the brain inflammatory responses are severely suppressed, as shown in mice with genetic deficiency of interleukin-6 (IL-6) or macrophage colony stimulating factor (M-CSF), the subsequent repair mechanisms and tissue remodelling may not occur or they are highly insufficient (Penkowa et al. 2002; Poulsen et al. 2005). Thus, cerebral inflammation can result in neurotoxicity with progressive neuron death or instead, it can mediate neuroprotection and CNS tissue repair.

Astroglia were previously considered as merely passive cells, which only provided structural support for the neurons, as to why they were named glia, which means ‘glue’. Now, astroglia are acknowledged to be of major importance for neuronal development, morphology and physiology including metabolism, synaptic transmission, neurovascular coupling and neuritogenesis (For reviews see: Darlington, 2005; Seifert et al. 2006; Sofroniew, 2005).

Reactive astrogliosis occurs in parallel to the acute pro-inflammatory responses of microglia/macrophages and lymphocytes (Carrasco et al. 2000; Espejo et al. 2005; Mhatre et al. 2004; Penkowa et al. 2005). Recently it has become clear that during brain disorders, the astrocytes are crucial for neuronal survival, functional recovery, synaptic plasticity and regeneration (Penkowa et al. 2004, 2006b; Sofroniew, 2005; Seifert et al. 2006).

In the brain, reactive astrocytes are the main source of neuroprotective factors with anti-inflammatory and/or antioxidant and/or anti-apoptotic roles, which explains why astroglia are crucial for neuronal survival, growth, and functioning (Mhatre et al. 2004; Penkowa et al. 2004, 2006b; Sofroniew 2005; Seifert et al. 2006). Accordingly, astrocytes provide important targets for the development of neuroprotective drugs. In order to develop new therapeutic strategies, the major components leading to delayed brain damage should each be targeted (Allan and Rothwell, 2003; Frederickson et al. 2004; Potts et al. 2006). This would require a polypharmaceutical strategy with drugs tailored towards the inflammatory response (like glucocorticoids, ibuprofen or minocycline), ROS formation (like SOD or glutathione peroxidase), and the excess of metals (metal chelator such as deferoxamine).

However, a more proficient approach would be to find a potential drug target that includes all three damage-causing aspects (monotherapy). Such target would ideally mediate anti-inflammatory and antioxidant actions and chelate (buffer) metal ions. In this regard, among the best known examples of such a drug target are MT-I+II, which prevent pro-inflammatory responses in the brain, oxidative stress, apoptotic cell death as well as they regulate and buffer the levels of metals.

In agreement with the presented MT-I+II actions, various brain disorders are more severe in MT-I+II deficient mice than in wildtype controls, whilst the clinical and histopathological symptoms are efficiently diminished by MT-I overexpression and exogenous MT-I or MT-II treatment (Aschner and West, 2005; Penkowa et al. 2006a, b). Hence, both endogenous and exogenous MT-I+II ameliorate neuroinflammation, neurodegeneration and neuronal apoptosis, as shown after brain injury, stroke, epilepsy, experimental autoimmune encephalomyelitis (EAE), amyotrophic lateral sclerosis (ALS), Parkinsons disease, pellagra-dementia, malnutrition, and encephalitis (Aschner and West, 2005; Chung et al. 2003, 2004; Ebadi et al. 2005a; Mocchegiani et al. 2005; Penkowa et al. 2006; Vasak, 2005; West et al. 2004). Generally, these data as well as studies of human brain tissue from neurological patients showed that apoptosis occurred in cells devoid of MT-I+II, while MT-I+II expressing cells were spared from apoptosis (Mocchegiani et al. 2005; Penkowa, 2006; West et al. 2004). This indicates that MT-I+II by some fundamental mechanisms overrule and prevent cell death, despite the fact that cells were exposed to pro-apoptotic signals.

Also, endogenous and exogenous MT-I+II can activate neuroglial stem cells as part of the brain repair responses. During EAE-demyelination, oligodendroglial progenitor cells are recruited by MT-I+II, whereby remyelination and recovery are promoted (Espejo et al. 2005; Penkowa and Hidalgo, 2003). Neural progenitor/stem cells are even more responsive to MT-I+II, which profoundly increase cell renewal and mobilization from the neurogenic zone as shown in the adult injured brain (Penkowa et al. 2006a).

In conclusion, MT-I+II can protect the brain from degeneration and cell death; while neuronal repair and functional recovery are improved. To this end, their potential for replacing lost neurons in the injured brain indicates a potential key role for MT-I+II in the future management of neurological diseases.

As listed in Table 1, MT-I+II mediate neuroprotection and regeneration, and thus, MT-I+II may provide drug targets for the development of pharmaceuticals against neurodegenerative diseases.

### Neoplasms

The MT-I+II roles in pathophysiological processes like pro-inflammatory signaling, ROS generation, cell degeneration and apoptosis, have led to many studies of MT-I+II expression during tumorigenesis (For review see: Theocharis et al. 2004). In fact, the antioxidant and anti-apoptotic roles of MT-I+II have in part been characterized due to such tumor studies, which mainly were carried out by exposing tumor cells with absent, unaltered or increased MT-I+II expression to a range of experimental anti-cancer therapies (Kennette et al. 2005; Klaassen et al. 1999; Kondo et al. 1997; Liu et al. 1999; Miles et al. 2000; Qu et al. 2006; Tekur and Ho, 2002).

Due to their cytoprotective actions, MT-I+II could be expected to defend neoplastic cells against the ROS-generating toxicity of radiation and chemotherapy. Accordingly, it is not surprising that MT-I+II expression levels were reported to be associated with tumor cell survival and resistance to treatment (For reviews see: Cai and Cherian, 2003; Cherian et al. 1994; Theocharis et al. 2003, 2004).

However, the vast majority of these papers have been descriptive, as they reported and correlated the pattern and extent of MT-I+II expression with tumor histology, grade, stage, invasiveness, and patients’ survival and prognosis (Cherian et al. 1994; Kondo et al. 1997; Miles et al. 2000; Poulsen et al. 2006; Qu et al. 2006; Tekur and Ho, 2002). These studies reported that MT-I+II expression levels are increased in a range of human tumors (e.g. tumors of the mammary gland, lung, naso-pharynx, colon, kidney, liver, salivary gland, testes, ovaries, prostate, thyroid gland, bone marrow hematopoietic cells and bladder). The increased MT-I+II levels have been correlated to malignancy and tumorigenesis, although it is based upon descriptive approaches lacking more mechanistic models and data. In fact, any direct causative roles of MT-I+II in oncogenic transformation remain to be identified.

Below, we will discuss some interesting findings on MT-I+II in relation to tumor biology.

### MT-I+II and clinical outcome - Part I

Increased expression levels of MT-I+II mRNA and protein have generally been coupled to cancer cell survival and resistance to various pro-apoptotic regimes including chemotherapy and radiation (Cherian et al. 2003; Miles et al. 2000; Kennette et al. 2005; Poulsen et al. 2006; Qu et al. 2006; Theocharis et al. 2003).

Hence, in malignant B-cell lymphomas and transitional cell carcinoma of the bladder, MT-I+II levels have been associated with worse outcome, as increased MT-I+II are inversely correlated to the patients’ survival (Poulsen et al. 2006; Yamasaki et al. 2006).

Also, increased MT-I+II levels are more frequent in high grade bladder tumors showing local invasion, relative to low grade, non-invasive tumors (Yamasaki et al. 2006).

Another study performed mRNA expression profiling by using Affymetrix genechips (micro-arrays) in diffuse large B-cell lymphomas, where it was shown that MT mRNA upregulation is an indicator of therapeutic failure (Poulsen et al. 2006). The same study also applied histopathological analyses that revealed MT-I+II expression in both the non-malignant cells (macrophages) and ín the malignant lymphocytes; although only the MT-I+II of the malignant cells were related to the clinical outcome. Hence, when MT-I+II expression was found in more than 20% of malignant cells, the patients experienced a significantly reduced 5-year survival (Poulsen et al. 2006).

In line with this, MT-I+II can confer cytoprotection against anti-neoplastic regimens, which typically cause ROS-induced DNA damage and break-down leading to apoptotic cell death (Abel and de Ruiter, 1989; Cai and Cherian, 2003; Tsangaris et al. 2000).

Also, MT-I+II are detected in gliomas, ependymomas and in the majority of meningiomas, although the expression levels are not consistently associated with clinical outcome (For reviews see: Klaassen et al. 1999; Theocharis et al. 2004). In fact, MT-I+II levels of brain gliomas were low when compared to MT-I+II expression of surrounding, reactive astrocytes, i.e. the non-malignant glia (Tews et al. 2000).

Accordingly, it is likely that a reported association between MT-I+II and clinical outcome in some cases might simply reflect the host defense response to tumor pathology, which includes induction of MT-I+II in order to endow cells (whether neoplastic or not) with potent survival mechanisms (Andrews, 2000; Tsangaris et al. 2000; Cherian et al. 2003; Kennette et al. 2005). It is also noteworthy that we lack data regarding the presumed role of MT-I+II in neoplastic transformation. So, a direct causative role of MT-I+II in pro-oncogenic events remains to be identified.

Nevertheless, tumor resistance to pro-apoptotic therapy represents a major problem in daily oncology. Since many reports did show a significant association between increased MT-I+II levels and tumor progression as well as clinical outcome, we will now discuss the possibility of differential MT-I+II regulation. If tumor cell-specific MT-I+II expression can be silenced without affecting the healthy bystander cells, such a strategy may provide a successful adjuvant to be used along anti-neoplastic regimens in order to prevent cell survival due to MT-I+II induction.

### Differential MT-I+II regulation

A differential and tissue-specific regulation of MT-I+II may alleviate some of the major problems with resistance to anti-cancer therapy. The optimum solution in this regard, would be to inhibit MT-I+II specifically in the malignant cells, and at the same time induce or not affect MT-I+II in the the healthy bystander tissue. Such a strategy may both counter the tumor resistance to treatment and the aspect of toxic side-effects, which are major obstacles to the curative treatment of cancer. Interestingly, the metal bismuth has been shown to induce MT-I+II in normal (non-malignant) tissue only, while malignant cells remain rather unaffected (Cherian et al. 1994, 2003). This may be due to the fact that bismuth accumulates in the healthy cells, while it is poorly taken up by transformed cells, although it is unclear how bismuth distinguishes between healthy versus malignant cells.

However, it was shown that bismuth affects different glycolytic enzymes and metabolites that have housekeeping and physiological roles (Magnusson et al. 2005). Such parameters and their signaling may indeed be disturbed in neoplastic cells, which could explain their poor import of bismuth.

To this end, Magnusson et al. (2005) propose that the bismuth mechanisms of action involve a “hypoxia-like” stress mimicking the molecular changes seen in normal cells exposed to ischemia. As tumor cells often show very early upregulation of hypoxia-induced, pro-angiogenic factors as part of their malignant transformation, these signaling pathways may already be perturbed in the tumor cells, which thereby have lost their import mechanism for bismuth (Cai and Cherian, 2003; Kennette et al. 2005; Kondo et al. 2004; Theocharis et al. 2003; Tsangaris et al. 2000).

Accordingly, bismuth may become an adjuvant that maintains or increases MT-I+II expression only in healthy cells, which thereby are defended against ROS-generation and cytotoxicity caused by radiation or chemotherapy. At the same time, the malignant cells likely remain unaffected by bismuth, as to why these cells may still be targeted by anti-neoplastic strategies.

Within this context, bismuth administration shows a promising potential and since it is already being used in the clinic, it may be more readily applicable as a pharmaceutical.

However, with new technologies and improved scientific equipment, we envisage that very soon other approaches will emerge. These may likely include new disciplines such as gene-directed techniques, small interference RNA-based regulation, tumor cell-specific transcriptomics, metabolomics including the potential to control metabolite degradation or turnover rate, and organ-targeted control of cell differentiation and cell replacement strategies.

### MT-I+II and clinical outcome - Part II

In this second part, we will briefly introduce some recent data, which indicate that increased MT-I+II levels are correlated to either better or unaltered prognosis, at least in some specific tumor tissues (Theocharis et al. 2003, 2004).

In human liver, MT-I+II expression is reduced in neoplastic cells as shown during carcinomas and adenocarcinoid metastases; whereas intense MT-I+II induction is found in the surrounding healthy cells (Cherian et al. 2003; Theocharis et al. 2003). This hepatic MT-I+II downregulation is considered to be tumor cell-specific, as MT-I+II are increased in non-malignant cirrhotic nodules, which are surrounding the malignant hepatocytes (Theocharis et al. 2004). Recently, a significant repression of MT-I mRNA was reported in primary hepatocellular carcinoma and in 7 different types of hepatocellular carcinoma cell lines (Chan et al. 2006). Hence, in human primary liver carcinomas, MT I transcripts were repressed by more than 100-fold, as shown by using gene expression profiling and qRT-PCR (Chan et al. 2006). The same study showed that MT-I mRNA was reduced in both early and later stages of hepatocellular cancer, whereby inactivation of MT-I might have an impact on tumor development and/or progression.

This expression pattern is also found in some head and neck cancers, renal neoplasms, sarcomas and breast adenocarcinomas, where increased MT-I+II are detected specifically in the healthy bystander cells (Dutsch-Wicherek et al. 2005; Ishii et al. 2001; Theocharis et al. 2004), which suggests that increased MT-I+II levels could simply be part of the normal cellular response to tumor pathology.

In colorectal cancer, positive MT-I+II expression is connected to a favourable prognosis and improved outcome, as MT-I+II progressively diminish during oncogenic transformation of healthy colorectal mucosa into adenomatous polyps and adenocarcinoma (Theocharis et al. 2004).

In other tissue types such as kidney and skeleton, MT-I+II may not influence tumor biology. Hence, the expression of MT-I+II was comparable in osteosarcoma patients and their healthy controls, and MT-I+II levels were not related to survival rate (Theocharis et al. 2004). Also, MT-I+II levels are not associated with histological type, stage of disease, or the prognosis of renal cell carcinomas (Mitropoulos et al. 2005). However, data obtained in kidney are not clear, since another study showed a 15-fold reduction in the MT-I+II levels of renal cancer cells relative to surrounding healthy cells (Ishii et al. 2001), while MT-I+II induction in renal neoplasms may also relate to a poor prognosis and a malignant behavior of the renal tumor, as discussed by Theocharis et al. (2004).

### MT-I+II in neoplasms: Friend or Foe?

From the current data, it is unknown whether MT-I+II are involved in malignant transformation, or if the observed changes in MT-I+II are epiphenomena caused by the general neoplastic pathology. To this end, it is likely of high importance to distinguish between MT-I+II expression in the malignant cells versus healthy bystander cells, as the latter may likely alter their MT-I+II in their general response to neoplastic pathology (Dutsch-Wicherek et al. 2005; Ishii et al. 2001; Poulsen et al. 2006).

Accordingly, increased MT-I+II levels in the healthy cells surrounding the tumor may represent a host defense mechanism as seen in other non-neoplastic pathologies.

Overall, an intricate variety of gene expression changes are seen during carcinogenesis and these include activation of proto-oncogenes, mitogens and genes critical to regulation of growth and survival of cells. During this, the cells require essential metals like Zn, which is needed for Zn-dependent transcription factors, enzymes, and Zn-finger proteins (Hidalgo et al. 2001, 2002; Vallee, 1995; Vasak, 2005). The altered Zn requirements alone, but also an altered gene expression profile may explain why MT-I+II expression has changed in some tumor cells. The fact that MT-I+II are rapidly induced by any kind of pathology or stress may also contribute to the MT-I+II increases found in some tumor cells. This indicates that increased MT-I+II levels are not necessarily reflecting causative roles for MT-I+II in tumorigenesis.

Moreover, the profound variation in MT-I+II expression between tumors may simply reflect general differences in the biological behavior of the neoplasms. Hence, tumors showing highly increased MT-I+II together with malignant behavior might represent tissues that in general show high protein turnover and/or high pro-angiogenic activity and/or proliferation rates. Even though such features may obviously provide tumor cells with resistance to radiation and chemotherapy (Cherian et al. 1994, 2003; Theocharis et al. 2003, 2004), they are not indicating mechanistic roles of MT-I+II in carcinogenesis.

In general, cell cultures and animals with genetic MT-I+II deficiency are more susceptible to radiation- and chemotherapy-induced cell death than wildtype controls or transgenic MT-I overexpressors (Andrews, 2000; Klaassen et al. 1999). In wildtype animals with intact MT-I+II genes, experimental Zn treatment rapidly induces MT-I+II and protects against cell death caused by anti-cancer therapy (Kennette et al. 2005). However, the Zn treatment gave the same protection against anti-cancer therapy in MT-I+II deficient mice, which suggests that resistance to anti-neoplastic treatment is due to other Zn-induced factors than MT-I+II (For review see: Klaassen et al. 1999).

Together, these data indicate that MT-I+II alone are not responsible for tumor cell-specific resistance to anti-neoplastic treatments.

Accordingly, it is impossible, at least at the moment, to define MT-I+II as biomarkers of prognosis or clinical outcome in relation to neoplasms.

## Figures and Tables

**Table 1:** 

**MT-I+II inhibitory actions**
MT-I+II scavenge and neutralize ROS
MT-I+II inhibit pro-inflammatory cytokines, macrophages and T-lymphocytes.
MT-I+II inhibit apoptosis (incl. cytochrome-c leakage, p53 levels, caspase activity)
**MT-I+II stimulatory actions**
MT-I+II augment anti-inflammatory and neuroprotective parameters in CNS
MT-I+II increase mitogens, apoptosis-inhibitory genes and growth/trophic factors
MT-I+II promote functional recovery including neurogenesis
**MT-I+II homeostatic regulation**
MT-I+II control and regulate toxic and essential metals
MT-I+II regulate NFkB concentration and activity

## References

[b1-bmi-2006-099] Abel J de Ruiter N 1989 Inhibition of hydroxyl-radical-generated DNA degradation by metallothionein Toxicol Lett 47 191 6 2545017 10.1016/0378-4274(89)90075-1

[b2-bmi-2006-099] Allan SM Rothwell NJ 2003 Inflammation in central nervous system injury Philos Trans R Soc Lond B Biol Sci 358 1669 77 14561325 10.1098/rstb.2003.1358 PMC1693261

[b3-bmi-2006-099] Andrews GK 2000 Regulation of metallothionein gene expression by oxidative stress and metal ions Biochem Pharmacol 59 95 104 10605938 10.1016/s0006-2952(99)00301-9

[b4-bmi-2006-099] Aschner M West AK 2005 The role of MT in neurological disorders J Alzheimers Dis 8 139 45 16308482 10.3233/jad-2005-8206

[b5-bmi-2006-099] Beattie JH Wood AM Newman AM 1998 Obesity and hyperleptinemia in metallothionein (-I and -II) null mice Proc Natl Acad Sci USA 95 358 63 9419380 10.1073/pnas.95.1.358 PMC18223

[b6-bmi-2006-099] Blindauer CA Sadler PJ 2005 How to hide zinc in a small protein Acc Chem Res 38 62 9 15654738 10.1021/ar030182c

[b7-bmi-2006-099] Cai L Cherian MG 2003 Zinc-metallothionein protects from DNA damage induced by radiation better than glutathione and copper- or cadmium-metallothioneins Toxicol Lett 136 193 8 12505272 10.1016/s0378-4274(02)00359-4

[b8-bmi-2006-099] Carrasco J Penkowa M Hadberg H 2000 Enhanced seizures and hippocampal neurodegeneration following kainic acid-induced seizures in metallothionein-I + II-deficient mice Eur J Neurosci 12 2311 22 10947810 10.1046/j.1460-9568.2000.00128.x

[b9-bmi-2006-099] Ceballos D Lago N Verdu E 2003 Role of metallothioneins in peripheral nerve function and regeneration Cell Mol Life Sci 60 1209 16 12861386 10.1007/s00018-003-3047-2 PMC11138647

[b10-bmi-2006-099] Chan KY Lai PB Squire JA 2006 Positional expression profiling indicates candidate genes in deletion hotspots of hepatocellular carcinoma Mod Pathol Sep 15 [Epub ahead of print] 10.1038/modpathol.3800674 16980951

[b11-bmi-2006-099] Chen Y Maret W 2001 Catalytic selenols couple the redox cycles of metallothionein and glutathione Eur J Biochem 268 3346 53 11389738 10.1046/j.1432-1327.2001.02250.x

[b12-bmi-2006-099] Cherian MG Apostolova MD 2000 Nuclear localization of metallothionein during cell proliferation and differentiation Cell Mol Biol, (Noisy -le-grand) 46 347 56 10774924

[b13-bmi-2006-099] Cherian MG Howell SB Imura N 1994 Role of metallothionein in carcinogenesis Toxicol Appl Pharmacol 126 1 5 8184419 10.1006/taap.1994.1083

[b14-bmi-2006-099] Cherian MG Jayasurya A Bay BH 2003 Metallothioneins in human tumors and potential roles in carcinogenesis Mutat Res 533 201 9 14643421 10.1016/j.mrfmmm.2003.07.013

[b15-bmi-2006-099] Chung RS Adlard PA Dittmann J 2004 Neuronglia communication: metallothionein expression is specifically up-regulated by astrocytes in response to neuronal injury J Neurochem 88 454 61 14690533 10.1046/j.1471-4159.2003.02193.x

[b16-bmi-2006-099] Chung RS Vickers JC Chuah MI 2003 Metallothionein-IIA promotes initial neurite elongation and postinjury reactive neurite growth and facilitates healing after focal cortical brain injury J Neurosci 23 3336 42 12716941 10.1523/JNEUROSCI.23-08-03336.2003 PMC6742325

[b17-bmi-2006-099] Chung RS West AK 2004 A role for extracellular metallothioneins in CNS injury and repair Neuroscience 123 595 9 14706772 10.1016/j.neuroscience.2003.10.019

[b18-bmi-2006-099] Crowthers KC Kline V Giardina C 2000 Augmented humoral immune function in metallothionein-null mice Toxicol Appl Pharmacol 166 161 72 10906280 10.1006/taap.2000.8961

[b19-bmi-2006-099] Darlington CL 2005 Astrocytes as targets for neuroprotective drugs Curr Opin Investig Drugs 6 700 3 16044665

[b20-bmi-2006-099] Davis SR Cousins RJ 2000 Metallothionein expression in animals: a physiological perspective on function J Nutr 130 1085 8 10801901 10.1093/jn/130.5.1085

[b21-bmi-2006-099] Dincer Z Haywood S Jasani B 1999 Immunocytochemical detection of metallothionein (MT1 and MT2) in copper-enhanced sheep brains J Comp Pathol 120 29 37 10098014 10.1053/jcpa.1998.0254

[b22-bmi-2006-099] Dutsch-Wicherek M Popiela TJ Klimek M 2005 Metallothionein stroma reaction in tumor adjacent healthy tissue in head and neck squamous cell carcinoma and breast adenocarcinoma Neuro Endocrinol Lett 26 567 74 16264399

[b23-bmi-2006-099] Ebadi M Brown-Borg H El Refaey H 2005a Metallothionein-mediated neuroprotection in genetically engineered mouse models of Parkinson’s disease Mol Brain Res 134 67 75 15790531 10.1016/j.molbrainres.2004.09.011 PMC3619407

[b24-bmi-2006-099] Ebadi M Sharma SK Ghafourifar P 2005b Peroxynitrite in the pathogenesis of Parkinson’s disease and the neuroprotective role of metallothioneins Methods Enzymol 396 276 98 16291239 10.1016/S0076-6879(05)96024-2

[b25-bmi-2006-099] Espejo C Penkowa M Demestre M 2005 Time-course expression of CNS inflammatory, neurodegenerative tissue repair markers and metallothioneins during experimental autoimmune encephalomyelitis Neuroscience 132 1135 49 16078373 10.1016/j.neuroscience.2005.01.057

[b26-bmi-2006-099] Feng W Cai J Pierce WM 2005 Metallothionein transfers zinc to mitochondrial aconitase through a direct interaction in mouse hearts Biochem Biophys Res Commun 332 853 8 15913554 10.1016/j.bbrc.2005.04.170

[b27-bmi-2006-099] Frederickson CJ Maret W Cuajungco MP 2004 Zinc and excito-toxic brain injury: a new model Neuroscientist 10 18 25 14987444 10.1177/1073858403255840

[b28-bmi-2006-099] Ghoshal K Jacob ST 2001 Regulation of metallothionein gene expression Prog Nucleic Acid Res Mol Biol 66 357 84 11051769 10.1016/s0079-6603(00)66034-8

[b29-bmi-2006-099] Giralt M Penkowa M Lago N 2002 Metallothionein-1+2 protect the CNS after a focal brain injury Exp Neurol 173 114 28 11771944 10.1006/exnr.2001.7772

[b30-bmi-2006-099] Gong YH Elliott JL 2000 Metallothionein expression is altered in a transgenic murine model of familial amyotrophic lateral sclerosis Exp Neurol 162 27 36 10716886 10.1006/exnr.2000.7323

[b31-bmi-2006-099] Haidara K Moffatt P Denizeau F 1999 Metallothionein induction attenuates the effects of glutathione depletors in rat hepatocytes Toxicol Sci 49 297 305 10416275 10.1093/toxsci/49.2.297

[b32-bmi-2006-099] Haq F Mahoney M Koropatnick J 2003 Signaling events for metallothionein induction Mutat Res 533 211 26 14643422 10.1016/j.mrfmmm.2003.07.014

[b33-bmi-2006-099] Haydon PG Carmignoto G 2006 Astrocyte control of synaptic transmission and neurovascular coupling Physiol Rev 86 1009 31 16816144 10.1152/physrev.00049.2005

[b34-bmi-2006-099] Hidalgo J Aschner M Zatta P 2001 Roles of the metallothionein family of proteins in the central nervous system Brain Res Bull 55 133 45 11470309 10.1016/s0361-9230(01)00452-x

[b35-bmi-2006-099] Hidalgo J Penkowa M Giralt M 2002 Metallothionein expression and oxidative stress in the brain Methods Enzymol 348 238 49 11885277 10.1016/s0076-6879(02)48642-9

[b36-bmi-2006-099] Ishii K Usui S Yamamoto H 2001 Decreases of metallothionein and aminopeptidase N in renal cancer tissues J Biochem (Tokyo) 129 253 8 11173527 10.1093/oxfordjournals.jbchem.a002852 PMC7109645

[b37-bmi-2006-099] Jiang LJ Maret W Vallee BL 1998 The glutathione redox couple modulates zinc transfer from metallothionein to zinc-depleted sorbitol dehydrogenase Proc Natl Acad Sci USA 95 3483 8 9520392 10.1073/pnas.95.7.3483 PMC19862

[b38-bmi-2006-099] Karin M Eddy RL Henry WM Haley LL Byers MG Shows TB 1984 Human metallothionein genes are clustered on chromosome 16 Proc Natl Acad Sci USA 81 5494 8 6089206 10.1073/pnas.81.17.5494 PMC391732

[b39-bmi-2006-099] Kägi JH Kojima Y 1987 Chemistry and biochemistry of metallothionein Experientia Suppl 52 25 61 2959513 10.1007/978-3-0348-6784-9_3

[b40-bmi-2006-099] Kelly EJ Palmiter RD 1996 A murine model of Menkes disease reveals a physiological function of metallothionein Nat Genet 13 219 22 8640230 10.1038/ng0696-219

[b41-bmi-2006-099] Kelly EJ Quaife CJ Froelick GJ 1996 Metallothionein I and II protect against zinc deficiency and zinc toxicity in mice J Nutr 126 1782 90 8683339 10.1093/jn/126.7.1782

[b42-bmi-2006-099] Kennette W Collins OM Zalups RK 2005 Basal and zinc-induced metallothionein in resistance to cadmium, cisplatin, zinc, and tertbutyl hydroperoxide: studies using MT knockout and antisense-downregulated MT in mammalian cells Toxicol Sci 88 602 13 16150881 10.1093/toxsci/kfi318

[b43-bmi-2006-099] Khatai L Goessler W Lorencova H 2004 Modulation of nitric oxide-mediated metal release from metallothionein by the redox state of glutathione in vitro Eur J Biochem 271 2408 16 15182356 10.1111/j.1432-1033.2004.04160.x

[b44-bmi-2006-099] Klaassen CD Choudhuri S McKim J.M. Jr 1994 In vitro and in vivo studies on the degradation of metallothionein Environ Health Perspect 102 Suppl 3 141 6 7843089 10.1289/ehp.94102s3141 PMC1567434

[b45-bmi-2006-099] Klaassen CD Liu J Choudhuri S 1999 Metallothionein: an intracellular protein to protect against cadmium toxicity Annu Rev Pharmacol Toxicol 39 267 94 10331085 10.1146/annurev.pharmtox.39.1.267

[b46-bmi-2006-099] Kohler LB Berezin V Bock E 2003 The role of metallothionein II in neuronal differentiation and survival Brain Res 992 128 36 14604781 10.1016/j.brainres.2003.08.049

[b47-bmi-2006-099] Kondo Y Himeno S Satoh M 2004 Citrate enhances the protective effect of orally administered bismuth subnitrate against the nephrotoxicity of cis-diamminedichloroplatinum Cancer Chemother Pharmacol 53 33 8 14530870 10.1007/s00280-003-0706-9

[b48-bmi-2006-099] Kondo Y Rusnak JM Hoyt DG 1997 Enhanced apoptosis in metallothionein null cells Mol Pharmacol 52 195 201 9271341 10.1124/mol.52.2.195

[b49-bmi-2006-099] Liu J Kimler BF Liu Y 1999 Metallothionein-I transgenic mice are not protected from gamma-radiation Toxicol Lett 104 183 7 10079052 10.1016/s0378-4274(98)00362-2

[b50-bmi-2006-099] Magnusson NE Larsen A Rungby J 2005 Gene expression changes induced by bismuth in a macrophage cell line Cell Tissue Res 321 195 210 15912405 10.1007/s00441-005-1103-y

[b51-bmi-2006-099] Maret W 2002 Optical methods for measuring zinc binding and release, zinc coordination environments in zinc finger proteins, and redox sensitivity and activity of zinc-bound thiols Methods Enzymol 348 230 7 11885276 10.1016/s0076-6879(02)48641-7

[b52-bmi-2006-099] Maret W Heffron G Hill HA 2002 The ATP/metallothionein interaction: NMR and STM Biochemistry 41 1689 94 11814364 10.1021/bi0116083

[b53-bmi-2006-099] Masters BA Kelly EJ Quaife CJ 1994 Targeted disruption of metallothionein I and II genes increases sensitivity to cadmium Proc Natl Acad Sci USA 91 584 88 8290567 10.1073/pnas.91.2.584 PMC42993

[b54-bmi-2006-099] Mhatre M Floyd RA Hensley K 2004 Oxidative stress and neuroinflammation in Alzheimer’s disease and amyotrophic lateral sclerosis: common links and potential therapeutic targets J Alzheimers Dis 6 147 57 15096698 10.3233/jad-2004-6206

[b55-bmi-2006-099] Michalska AE Choo KH 1993 Targeting and germ-line transmission of a null mutation at the metallothionein I and II loci in mouse Proc Natl Acad Sci USA 90 8088 92 8367468 10.1073/pnas.90.17.8088 PMC47293

[b56-bmi-2006-099] Miles AT Hawksworth GM Beattie JH 2000 Induction, regulation, degradation, and biological significance of mammalian metallothioneins Crit Rev Biochem Mol Biol 35 35 70 10755665 10.1080/10409230091169168

[b57-bmi-2006-099] Min KS Tanaka N Horie T 2005 Metallothionein-enriched hepatocytes are resistant to ferric nitriloacetate toxicity during conditions of glutathione depletion Toxicol Lett 158 108 15 16039399 10.1016/j.toxlet.2005.03.001

[b58-bmi-2006-099] Mitropoulos D Kyroudi-Voulgari A Theocharis S 2005 Prognostic significance of metallothionein expression in renal cell carcinoma World J Surg Oncol 3 5 15655072 10.1186/1477-7819-3-5 PMC545945

[b59-bmi-2006-099] Mocchegiani E Bertoni-Freddari C Marcellini F 2005 Brain, aging and neurodegeneration: role of zinc ion availability Prog Neurobiol 75 367 90 15927345 10.1016/j.pneurobio.2005.04.005

[b60-bmi-2006-099] Nagano S Satoh M Sumi H 2001 Reduction of metallothioneins promotes the disease expression of familial amyotrophic lateral sclerosis mice in a dose-dependent manner Eur J Neurosci 13 1363 70 11298796 10.1046/j.0953-816x.2001.01512.x

[b61-bmi-2006-099] Ostrakhovitch EA Olsson PE Jiang S 2006 Interaction of metallothionein with tumor suppressor p53 protein FEBS Lett 580 1235 8 16442532 10.1016/j.febslet.2006.01.036

[b62-bmi-2006-099] Palmiter RD 1998 The elusive function of metallothioneins Proc Natl Acad Sci USA 95 8428 30 9671693 10.1073/pnas.95.15.8428 PMC33872

[b63-bmi-2006-099] Penkowa M 2006 Metallothioneins are multipurpose neuroprotectants during brain pathology FEBS J 273 1857 70 16640552 10.1111/j.1742-4658.2006.05207.x

[b64-bmi-2006-099] Penkowa M Cáceres M Borup R 2006a Novel Roles for Metallothionein-I+II (MT-I+II) in Defense Responses, Neurogenesis and Tissue Restoration after Traumatic Brain Injury: Insights from global gene expression profiling in wildtype and MT-I+II knockout mice J Neurosci Res10.1002/jnr.21043 16941634

[b65-bmi-2006-099] Penkowa M Carrasco J Giralt M 1999 CNS wound healing is severely depressed in metallothionein I- and II-deficient mice J Neurosci 19 2535 45 10087067 10.1523/JNEUROSCI.19-07-02535.1999 PMC6786080

[b66-bmi-2006-099] Penkowa M Florit S Giralt M 2005 Metallothionein reduces central nervous system inflammation, neurodegeneration, and cell death following kainic acid-induced epileptic seizures J Neurosci Res 79 522 34 15614785 10.1002/jnr.20387

[b67-bmi-2006-099] Penkowa M Hidalgo J 2001 Metallothionein treatment reduces proinflammatory cytokines IL-6 and TNF-alpha and apoptotic cell death during experimental autoimmune encephalomyelitis (EAE) Exp Neurol 170 1 14 11421579 10.1006/exnr.2001.7675

[b68-bmi-2006-099] Penkowa M Hidalgo J 2003 Treatment with metallothionein prevents demyelination and axonal damage and increases oligodendrocyte precursors and tissue repair during experimental autoimmune encephalomyelitis J Neurosci Res 72 574 86 12749022 10.1002/jnr.10615

[b69-bmi-2006-099] Penkowa M Moos T Carrasco J 1999 Strongly compromised inflammatory response to brain injury in interleukin-6-deficient mice Glia 25 343 57 10028917

[b70-bmi-2006-099] Penkowa M Poulsen C Carrasco J 2002 M-CSF deficiency leads to reduced metallothioneins I and II expression and increased tissue damage in the brain stem after 6-aminonicotinamide treatment Exp Neurol 176 308 21 12359172 10.1006/exnr.2002.7968

[b71-bmi-2006-099] Penkowa M Quintana A Carrasco J 2004 Metallothionein prevents neurodegeneration and central nervous system cell death after treatment with gliotoxin 6-aminonicotinamide J Neurosci Res 77 35 53 15197737 10.1002/jnr.20154

[b72-bmi-2006-099] Penkowa M Tio L Giralt M 2006b Specificity and divergence in the neurobiologic effects of different metallothioneins after brain injury J Neurosci Res 83 974 84 16493670 10.1002/jnr.20790

[b73-bmi-2006-099] Potashkin JA Meredith GE 2006 The role of oxidative stress in the dysregulation of gene expression and protein metabolism in neurodegenerative disease Antioxid Redox Signal 8 144 51 16487048 10.1089/ars.2006.8.144

[b74-bmi-2006-099] Potts MB Koh SE Whetstone WD 2006 Traumatic injury to the immature brain: inflammation, oxidative injury, and iron-mediated damage as potential therapeutic targets NeuroRx 3 143 53 16554253 10.1016/j.nurx.2006.01.006 PMC3593438

[b75-bmi-2006-099] Poulsen CB Penkowa M Borup R 2005 Brain response to traumatic brain injury in wild-type and interleukin-6 knockout mice: a microarray analysis J Neurochem 92 417 32 15663489 10.1111/j.1471-4159.2004.02877.x

[b76-bmi-2006-099] Poulsen CB Borup R Borregaard N 2006 Prognostic significance of metallothionein in B-cell lymphomas Blood In Press. (Blood. 2006 Jul 25[Epub ahead of print]) 10.1182/blood-2006-04-015305 16868254

[b77-bmi-2006-099] Qu W Fuquay R Sakurai T 2006 Acquisition of apoptotic resistance in cadmium-induced malignant transformation: specific perturbation of JNK signal transduction pathway and associated metallothionein overexpression Mol Carcinog 45 561 71 16568437 10.1002/mc.20185

[b78-bmi-2006-099] Romero-Isart N Vasak M 2002 Advances in the structure and chemistry of metallothioneins J Inorg Biochem 88 388 96 11897355 10.1016/s0162-0134(01)00347-6

[b79-bmi-2006-099] Searle PF Davison BL Stuart GW 1984 Regulation, linkage, and sequence of mouse metallothionein I and II genes Mol Cell Biol 4 1221 30 6095054 10.1128/mcb.4.7.1221-1230.1984 PMC368902

[b80-bmi-2006-099] Seifert G Schilling K Steinhauser C 2006 Astrocyte dysfunction in neurological disorders: a molecular perspective Nat Rev Neurosci 7 194 206 16495941 10.1038/nrn1870

[b81-bmi-2006-099] Sofroniew MV 2005 Reactive astrocytes in neural repair and protection Neuroscientist 11 400 7 16151042 10.1177/1073858405278321

[b82-bmi-2006-099] Stankovic RK 2005 Atrophy of large myelinated axons in metallothionein-I, II knockout mice Cell Mol Neurobiol 25 943 53 16133945 10.1007/s10571-005-4960-8 PMC11529569

[b83-bmi-2006-099] Tamai KT Gralla EB Ellerby LM 1993 Yeast and mammalian metallothioneins functionally substitute for yeast copper-zinc superoxide dismutase Proc Natl Acad Sci USA 90 8013 7 8367458 10.1073/pnas.90.17.8013 PMC47278

[b84-bmi-2006-099] Tekur S Ho SM 2002 Ribozyme-mediated downregulation of human metallothionein II(a) induces apoptosis in human prostate and ovarian cancer cell lines Mol Carcinog 33 44 55 11807957 10.1002/mc.10017

[b85-bmi-2006-099] Tews DS Nissen A Kulgen C 2000 Drug resistance-associated factors in primary and secondary glioblastomas and their precursor tumors J Neurooncol 50 227 37 11263502 10.1023/a:1006491405010

[b86-bmi-2006-099] Theocharis SE Margeli AP Klijanienko JT 2004 Metallothionein expression in human neoplasia Histopathology 45 103 18 15279628 10.1111/j.1365-2559.2004.01922.x

[b87-bmi-2006-099] Theocharis SE Margeli AP Koutselinis A 2003 Metallothionein: a multifunctional protein from toxicity to cancer Int J Biol Markers 18 162 9 14535585 10.1177/172460080301800302

[b88-bmi-2006-099] Thornalley PJ Vasak M 1985 Possible role for metallothionein in protection against radiation-induced oxidative stress. Kinetics and mechanism of its reaction with superoxide and hydroxyl radicals Biochim Biophys Acta 827 36 44 2981555 10.1016/0167-4838(85)90098-6

[b89-bmi-2006-099] Tsangaris GT Vamvoukakis J Politis I 2000 Metallothionein expression prevents apoptosis. II: Evaluation of the role of metallothionein expression on the chemotherapy-induced apoptosis during the treatment of acute leukemia Anticancer Res 20 4407 11 11205280

[b90-bmi-2006-099] Vallee BL 1995 The function of metallothionein Neurochem Int 27 23 33 7655345 10.1016/0197-0186(94)00165-q

[b91-bmi-2006-099] Vasak M 2005 Advances in metallothionein structure and functions J Trace Elem Med Biol 19 13 7 16240666 10.1016/j.jtemb.2005.03.003

[b92-bmi-2006-099] Wanpen S Govitrapong P Shavali S 2004 Salsolinol, a dopamine-derived tetrahydroisoquinoline, induces cell death by causing oxidative stress in dopaminergic SH-SY5Y cells, and the said effect is attenuated by metallothionein Brain Res 1005 67 76 15044066 10.1016/j.brainres.2004.01.054

[b93-bmi-2006-099] West AK Chuah MI Vickers JC 2004 Protective role of metallothioneins in the injured mammalian brain Rev Neurosci 15 157 66 15357139 10.1515/revneuro.2004.15.3.157

[b94-bmi-2006-099] Yamasaki Y Smith C Weisz D 2006 Metallothionein expression as prognostic factor for transitional cell carcinoma of bladder Urology 67 530 5 16504266 10.1016/j.urology.2005.09.033

[b95-bmi-2006-099] Ye B Maret W Vallee BL 2001 Zinc metallothionein imported into liver mitochondria modulates respiration Proc Natl Acad Sci USA 98 2317 22 11226237 10.1073/pnas.041619198 PMC30136

